# Identification of Heme Oxygenase 1 (HO-1) as a Novel Negative Regulator of Mobilization of Hematopoietic Stem/Progenitor Cells

**DOI:** 10.1007/s12015-014-9547-7

**Published:** 2014-08-03

**Authors:** Marcin Wysoczynski, Janina Ratajczak, Daniel Pedziwiatr, Gregg Rokosh, Roberto Bolli, Mariusz Z. Ratajczak

**Affiliations:** 1 Institute of Molecular Cardiology, University of Louisville, Louisville, KY USA; 2 Stem Cell Institute at James Graham Brown Cancer Center, University of Louisville, 500 S. Floyd Street, Rm. 107, Louisville, KY 40202 USA; 3 Department of Physiology, Pomeranian University of Medicine, 70-111 Szczecin, Poland

**Keywords:** Stem cell mobilization, Heme oksygenase-1, Complement Cascade, SDF-1

## Abstract

Activation of complement cascade (ComC) play and important role in mobilization of hematopoietic stem/progenitor cells (HSPCs) from bone marrow (BM) into peripheral blood (PB). While there are vast experimental data on the mechanisms and factors that induce or promote mobilization of HSPCs, there is relatively less data on negative regulators of this process. We demonstrate for the first time that heme oxygenase-1 (HO-1) that has a well-documented anti-inflammatory potential plays an important and heretofore unrecognized role in retention of HSPCs in BM niches by i) modulating negatively activation of mobilization promoting ComC, ii) maintaining stromal derived factor-1 (SDF-1) level in the BM microenvironment and iii) attenuating chemotactic responsiveness of HSPCs to SDF-1 and sphingosine-1 phosphate (S1P) gradients in PB. Furthermore, our data showing a positive mobilizing effect by a non-toxic small-molecule inhibitor of HO-1 (SnPP) suggest that blockade of HO-1 would be a promising strategy to facilitate mobilization of HSPCs. Further studies are also needed to evaluate better the molecular mechanisms responsible for the potential effect of HO-1 in homing of HSPCs after transplantation.

**Electronic supplementary material:**

The online version of this article (doi:10.1007/s12015-014-9547-7) contains supplementary material, which is available to authorized users.

## Introduction

While there are vast experimental data in the literature on the mechanisms and factors that induce or promote mobilization of hematopoietic stem/progenitor cells (HSPCs) from bone marrow (BM) into peripheral blood (PB), there is relatively less data on negative regulators of this process. Inhibitory effects on mobilization have been described mainly for serine protease inhibitors (serpins) [1] and tissue inhibitors of metalloproteinases (TIMPs) [2].

It is well known that HSPCs are retained in BM niches due to interaction between the chemokine CXCR4 receptor and very late antigen-4 (VLA-4, also known as α_4_β_1_ integrin), which are expressed on the surface of HSPCs. Their respective ligands, α-chemokine stromal-derived growth factor-1 (SDF-1) and vascular adhesion molecule-1 (VCAM-1, also known as CD106), are expressed by cells in the BM stem cell niches (e.g., osteoblasts and fibroblasts) [3–6]. HSPCs circulate under steady-state conditions at detectable levels in the PB, and their number increases in PB in response to i) systemic or local inflammation, ii) stress, iii) tissue/organ injury, iv) strenuous exercise, and v) certain pharmacological agents such as granulocyte colony-stimulating factor (G-CSF) or the CXCR4 small-molecule antagonist AMD3100 [3–6]. All these processes trigger activation of complement cascade (ComC) [5].

Harvested from PB, pharmacologically mobilized HSPCs are a convenient source of cells for hematopoietic transplantation. It is well known that mobilization induces a proteolytic microenvironment in BM, and the mobilization process is promoted, at least in part, by the effect of proteolytic enzymes on attenuating SDF-1–CXCR4 and VLA-4–VCAM-1 interactions in the BM microenvironment [3–6]. Specifically, several proteolytic enzymes involved in mobilization of HSPCs are released from activated granulocytes and monocytes residing in BM. However, the fact that mice with knockouts of proteolytic enzymes, such as MMP-9, cathepsin G, and elastase, are normal mobilizers [7] indicates that this list is incomplete and suggests the involvement of other proteolytic enzymes secreted by BM-residing cells or even, as we recently proposed, the involvement of proteolytic cascades such as the complement cascade (ComC), coagulation cascade (CoaC), and fibrynolytic cascade (FibC) [8]. All these ancient proteolytic cascades are activated in response to stress situations, inflammation, and administration of mobilizing agents (e.g., G-CSF and AMD3100) [5].

In previous work, we reported a crucial role for the fifth protein component of ComC (C5) in mobilization of HSPCs [9]. C5-deficient mice are very poor mobilizers, and to explain this, we postulated that C5 cleavage fragments (C5a and C5b) are essential for egress of HSPCs from BM into PB. While C5a anaphylatoxin i) activates neutrophils and monocytes in the BM microenvironment to secrete proteolytic enzymes, ii) permeabilizes endothelium, and iii) chemoattracts neutrophils and monocytes into PB [9], the C5b-C9 complex (also known as membrane attack complex, MAC) releases from erythrocytes and platelets sphingosine-1-phosphate (S1P), which is a major chemoattractant for HSPCs in PB [10]. Therefore, we proposed that innate immunity (e.g., the functions of granulocytes and activated ComC) and the accompanying inflammatory process is a trigger for egress of HSPCs from BM into PB [5].

Since activated ComC is a trigger for mobilization and induces a proteolytic environment in BM, oxidative stress, activation of platelets, and damage to erythrocyte membranes, we became interested in mechanisms that control and attenuate this process. Heme oxygenase 1 (HO-1) is an inducible stress-responsive enzyme that not only catalyzes the degradation of heme (e.g., released from erythrocytes exposed to MAC) but also plays an important function in various physiological and pathophysiological states associated with cellular stress such as ischemic/reperfusion injury [11]. HO-1 has a well-documented anti-inflammatory potential and inhibits ComC-dependent inflammation by upregulating as reported expression of ComC inhibitors CD55 and CD59 on endothelial cells [12], and HO-1 deficiency in humans and mice results in vulnerability to stressful injury [11]. Interestingly, it has also been reported that HO-1 directly regulates the expression of SDF-1 [13], which, as mentioned above, provides a major retention signal for HSPCs in BM niches [3–6]. Moreover, HO-1 has been reported to have negative effect on adhesion and migration of neutrophils in acute inflammation [14]. Neutrophils are first cells that egress BM during mobilization and as we demonstrated are crucial to pave a way for HSPCs across BM-blood barrier [9].

To address the role of HO-1 in retention and mobilization of HSPCs in BM, we performed mobilization studies in HO-1 knockout mice (HO-1^+/−^ and HO-1^−/−^).

## Material and Methods

### Animals

In our studies we employed wild-type (+/+), heterozygous (+/−), or homozygous null (−/−) mice for targeted disruption of HO-1 [15]. These mice were maintained on a 129Sv × BALB/c genetic background, and littermates were used for the studies [15]. For some experiments additional mice C57BL/6 were purchased from Jackson Laboratories (Bar Harbor, ME). Animal studies were approved by the Animal Care and Use Committee of the University of Louisville (Louisville, KY).

### Mobilization

Mice were mobilized by subcutaneous injection of 100 μg/kg human G-CSF (Amgen, Thousand Oaks, CA) daily for 3 or 6 days; or single intraperitoneal injection of AMD3100 5 ug/kg (Sigma, St. Louis, MO). Six hours after the last G-CSF injection or 2 h post AMD3100 injection, PB was obtained from the vena cava (with a 25-gauge needle and 1-mL syringe containing 250 U heparin). MNC cells were obtained by hypotonic lysis of red blood cells in BD Pharm Lyse buffer (BD Biosciences, San Jose, CA) as described [8–10]. In some mobilization protocols the HO-1 inhibitor protoporphyrin IX (SnPP-IX) (Porphyrin Products, Logan, UT, USA) was injected intraperitoneally (30 mg/kg) one hour before G-CSF or AMD3100 injections.

### WBC Counts

Fifty microliters of PB was taken from the retro-orbital plexus of the mice and collected into microvette EDTA-coated tubes (Sarstedt Inc., Newton, NC). Samples were run within 2 h of collection on a HemaVet 950 (Drew Scientific Inc., Oxford, CT) as described [8–10].

### Colony Forming Unit-Granulocytes/Macrophage (CFU-GM) Assay for Circulating in PB HSPCs

Red blood cells (RBCs) were lysed with BD Pharm Lyse buffer (BD Biosciences, San Jose, CA). Nucleated cells from PB were subsequently washed twice and used for CFU-GM colonies, cells were resuspended in human methylcellulose base media provided by the manufacturer (R&D Systems, Inc., Minneapolis, MN) supplemented with 25 ng/ml recombinant murine granulocyte macrophage colony-stimulating factor (mGM-CSF) and 10 ng/ml recombinant murine interleukin-3 (mIL-3; Millipore, Billerica, MA). Cultures were incubated for 7 days, at which time they were scored for the number of CFU-GM colonies under an inverted microscope as described [8–10]. We cultured 1 × 10^6^ PBMNC/dish and final results are recalculated based on number of PBMNC/1 μl of peripheral blood.

### Fluorescence-Activated Cell Sorting (FACS) Analysis

BMNC staining was performed in medium containing 2 % fetal bovine serum (FBS). All monoclonal antibodies (mAbs) were added at saturating concentrations and the cells were incubated for 30 min on ice, washed twice, resuspended in staining solution at a concentration of 5 × 10^6^ cells/ml, and subjected to analysis using an LSR II (Becton Dickinson, Mountainview, CA). The following anti-mouse Abs were used to detect fluorescein isothiocyanate (FITC)-anti-CD117 (c-Kit) (clone 2B8; BioLegend, San Diego, CA) and Phycoerythrin (PE)-Cy5 anti-mouse Ly-6A/E (Sca-1) (clone D7; eBioscience™, San Diego, CA). All anti-mouse lineage markers (Lin) were conjugated by PE and purchased from BD Biosciences: anti-CD45R/B220 (clone RA3-6B2); anti-Gr-1 (clone RB6-8C5); anti-T-cell receptor β (TCRβ; clone H57-597); anti-TCRγδ (clone GL3); anti-CD11b (clone M1/70); anti-Ter-119 (clone TER-119); and anti-CD34 (clone RAM34) as described [8–10].

### Evaluation of HSPC Mobilization

The following formula was used for evaluation of circulating CFU-GM and Sca-1+/c-Kit+/Lin−/CD34− (SKL CD34−) cells: {[number of white blood cells (WBCs) × number of CFU-GM colonies]/number of WBCs plated = number of CFU-GM per microliter of PB} and {(number of WBCs × number of SKL CD34− cells)/number of gated WBCs = number of SKL cells per microliter of PB}, respectively [8–10]. We cultured 1 × 10^6^ PBMNC/dish and final results are recalculated based on number of PBMNC/1 μl of peripheral blood.

### Colony-Forming Assays

To evaluate the number of congenic progenitor cells BMMNC were resuspended in methylcellulose base media provided by the manufacturer (R&D Systems, Inc., Minneapolis, MN) supplemented with granulocyte macrophage colony-stimulating factor (GM-CSF, 25 ng/ml) plus interleukin-3 (IL-3, 10 ng/ml) for colony forming units (CFU) granulocyte/macrophage (GM), erythropoietin (EPO, 5 unit/ml, Stem Cell Tech) plus stem cell factor (SCF, 5 ng/ml) for burst-forming units (BFU-E), and thrombopoietin (TPO, 100 ng/ml) for CFU-megakaryocytes (Megs). All growth factors were purchased from the same company unless otherwise mentioned. Cultures were incubated for 7 days, at which time they were scored under an inverted microscope for the number of each colonies as described [10].

### Real-Time Quantitative Reverse-Transcription PCR

Total RNA was isolated from HO-1^+/+^ (WT), HO-1^+/−^ and HO-1^−/−^ mice BMMNC or BM stroma cells with the RNeasy kit (Qiagen, Valencia, CA). The RNA was reverse transcribed with MultiScribe reverse transcriptase, oligo(dT), and random-hexamer primer mix (Applied Biosystems, Foster City, CA). PCR was performed at 2 cycles of 2 min at 95 °C, 1 min at 60 °C, and 1 min at 72 °C; 36 cycles of 30 s at 95 °C, 1 min at 60 °C, and 1 min at 72 °C; and 1 cycle of 10 min at 72 °C. Quantitative assessment of mRNA levels was done by real-time reverse transcription PCR (RT-PCR) on an ABI 7500 instrument with Power SYBR Green PCR Master Mix reagent. Real-time conditions were as follows: 95 °C (15 s), 40 cycles at 95 °C (15 s), and 60 °C (1 min). We employed following primers: CXCR4 (forward): GTA CCG GCT GCA CCT GTC A, CXCR4 (reverse): CAA AAA TTT CCC AAA GTA CCA G, SDF-1 (forward): TCT GCA TCA GTG ACG GTA A and SDF-1 (reverse): GAG TGT TGA GGA TTT TCA G, β-2D (forward): TCC AGC TGT TGG AAG TTT AAA AAG T β-2D (reverse): AGG ACA AAT GGC TCT GAC ACA GT. According to melting point analysis, only one PCR product was amplified under these conditions. The relative quantity of a target, normalized to the endogenous β2-microglobulin gene as control and relative to a calibrator, is expressed as 2^−ΔΔCt^ (fold difference), where Ct is the threshold cycle, ΔCt = (Ct of target genes) − (Ct of the endogenous control gene, β2-microglobulin), and ΔΔCt = (ΔCt of samples for target gene) − (ΔCt of calibrator for the target gene) as described [10].

### Trans-Well Migration Assay

The trans-well migration assay was performed as described elsewhere [10]. Briefly, unless otherwise indicated, murine RBC-lysed BMNCs were resuspended in assay media (RPMI containing 0.5 % BSA) and equilibrated for 10 min at 37 °C. Assay medium (650 μl) containing test reagents was added to the lower chambers of a Costar Trans-well plate (Costar Corning, Cambridge, MA, USA). Aliquots of cell suspension (5 × 10^5^ cells/100 μl) were loaded onto the upper chambers with 5 μm-pore filters, then incubated for 3 h (37 °C, 5 % CO_2_). Cells from the lower chambers were scored using FACS analysis for migration of BMNCs to test reagents and plated in CFU-GM colony assays as described [10].

### Adhesion HSPC to BM Stroma

BMMNC cells from WT and HO-1 deficient mice were tested for adhesion to WT or HO-1 deficient BM stroma cells as described [16]. In our assay 10^5^ BMMNC cells of each genotype were plated over stroma cells. After 30 min of adhesion, non-adherent cells were discarded from the adhesion cultures and the cells in the wells were trypsinzed and subsequently plated in methylocellulose cultures stimulated to grow CFU-GM colonies as described [16]. Since marrow fibroblasts were irradiated before the adhesion assay, they did not grow after plating in methylcellulose.

### Plasma Concentration of C5b-C9 (MAC Complex)

The concentration of C5b-C9 was measured by employing the commercially available, highly sensitive ELISA kit K-ASSAY (Kamiya Biomedical Company, Seattle, Wa), according to the manufacturer’s protocol as described [8]. For analysis peripheral blood from C3 −/− mice was collected at day sixth of G-CSF-induced mobilization by the retro-orbital plexus bleeding into cold microvette EDTA-coated tubes (Sarstedt Inc., Newton, NC). Subsequently, blood was centrifuged 2000 × g for 20 min in 4 °C to obtain plasma.

## SDF-1 Measurement Assay

SDF-1 levels were evaluated in conditioned media from BM stroma cells and in PB plasma by sandwich ELISA using the commercially available ELISA system (R&D Systems, Minneapolis, MN, USA) as described [10].

### Statistics

All results were presented as mean ± SD. Statistical analysis of the data was done using Student’s *t* test for unpaired samples, with *p* < 0.05 considered significant.

## Results and Discussion

**Fig. 1:**
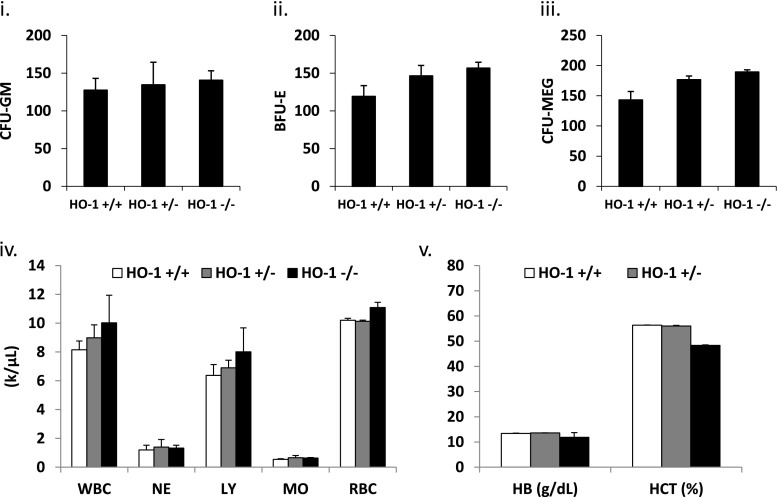
HO-1 does not affect hematological homeostasis. Bone marrow of WT (HO-1^+/+^), HO-1^+/−^ and HO-1^−/−^ mice was isolated and evaluated for number of CFU-GM (**i**), BFU-E (**ii**) and CFU-MEG (**iii**) in clonogenic in vitro assays. Peripheral blood parameters were evaluated by Hemavet and indicated no differences in number of white blood cells (WBC), neutrophils (NE), lymphocytes (LY), monocytes (MO) and red blood cells (RBC) (**iv**). HO-1 deficiency had no effect on hemoglobin (HB) content and hematocrit (HCT) (**v**). Data represent at average of at least eight mice per experimental group

To address the role of HO-1 in retention and mobilization of HSPCs in BM, we employed as an experimental model HO-1 knockout mice (HO-1^+/−^ and HO-1^−/−^) [15]. It has been reported that these animals with advancing age develop anemia [17]. However we noticed that HO-1^+/−^ and HO-1^−/−^ mice at the age of 6–8 weeks maintained in our colony still have the same number of hematopoietic progenitors in peripheral blood and peripheral blood counts as wild type (WT) littermates (Fig. 1). Therefore, since, this equivalence changes with advancing age we performed our mobilization studies employing mice that were 6–8 weeks old.

**Fig. 2:**
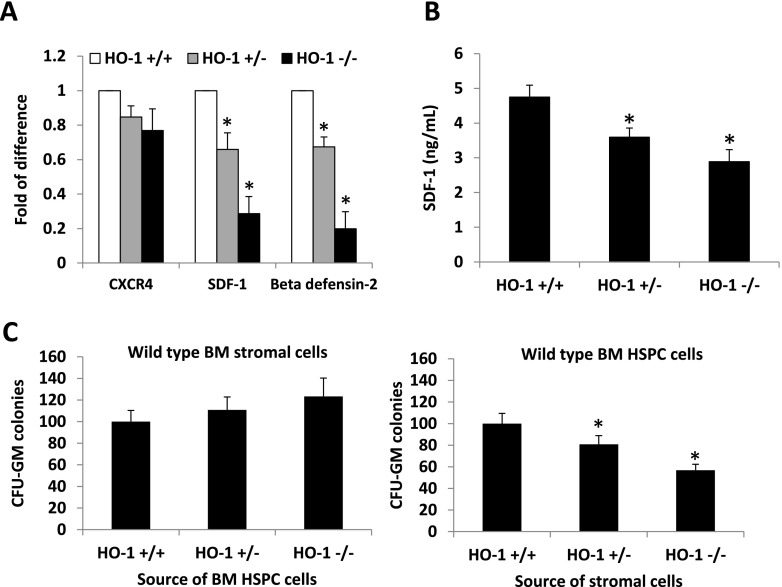
HO-1 deficiency reduces adhesive potential of bone marrow stromal cells. **Panel a** - Bone marrow mononuclear cells (BMMNC) and BM stromal cells were derived from bone marrow of HO-1^+/+^ (WT), HO-1^+/−^ and HO-1^−/−^ mice. Total RNA was isolated and quantitative real-time PCR was performed to determine expression of CXCR4 in BMMNC and SDF-1 and beta defensine-2 expression in BM stroma cells. * *p* < 0.05. **Panel b** – ELISA results demonstrating decrease in SDF-1 level in conditioned media harvested from BM stroma cell cultures from HO-1 deficient animals. * *p* < 0.05. **Panel c** - HO-1 does not affect adhesion of CFU-GMs to stromal cells isolated from wild type mice (*left*), however wild type CFU-GM have impaired adhesion to stromal cells isolated from HO-1+/− and HO-1−/− mice in comparison to HO-1+/+ (WT) (*right*). * *p* < 0.05. Number of CFU-GM colonies that adhered to stroma cells in left and right panel were assumed to be 100 %. Data represent average of three independent experiments (**a**–**c**)

Based on report that HO-1 directly regulates the expression of SDF-1 in ischemic heart [13], we employed RQ-PCR, and found that HO-1 deficiency results in a significant decrease in expression of SDF-1 in BM stromal cells. We also noticed decrease in expression of β2-defensin (β2-D) that is a modulator of responsiveness of HSPCs to SDF-1 gradient [5]. At the same time expression of CXCR4 that is SDF-1 binding receptor in BM mononuclerar cells (BMMNC) was unaffected (Fig. 2a). A decrease in SDF-1 at mRNA (Fig. 2a) and at protein level in bone marrow stroma cells (Fig. 2b) corroborates the observation that HO-1 regulates the SDF-1 level in myocardium [13]. This observation together with the decrease in expression of β2-D, which positively modulates the responsiveness of HSPCs to an SDF-1 gradient [5], indicates a potential defect in retention of HSPCs in BM niches in HO-1-deficient mice. The existence of this defect was further supported by defective adhesion of WT HSPCs in stromal cells of HO^−/−^ mice (Fig. 2c). Nevertheless, since HO-1 deficient mice have in steady state conditions a similar number of circulating SKL and CFU-GM cells in PB as WT littermates indicates that at age of 6–8 weeks retention of HSPCs in BM in mutant animals is not affected and compensated by other BM retention axes (e.g., VLA-4 – VCAM-1) [3–7].

**Fig. 3:**
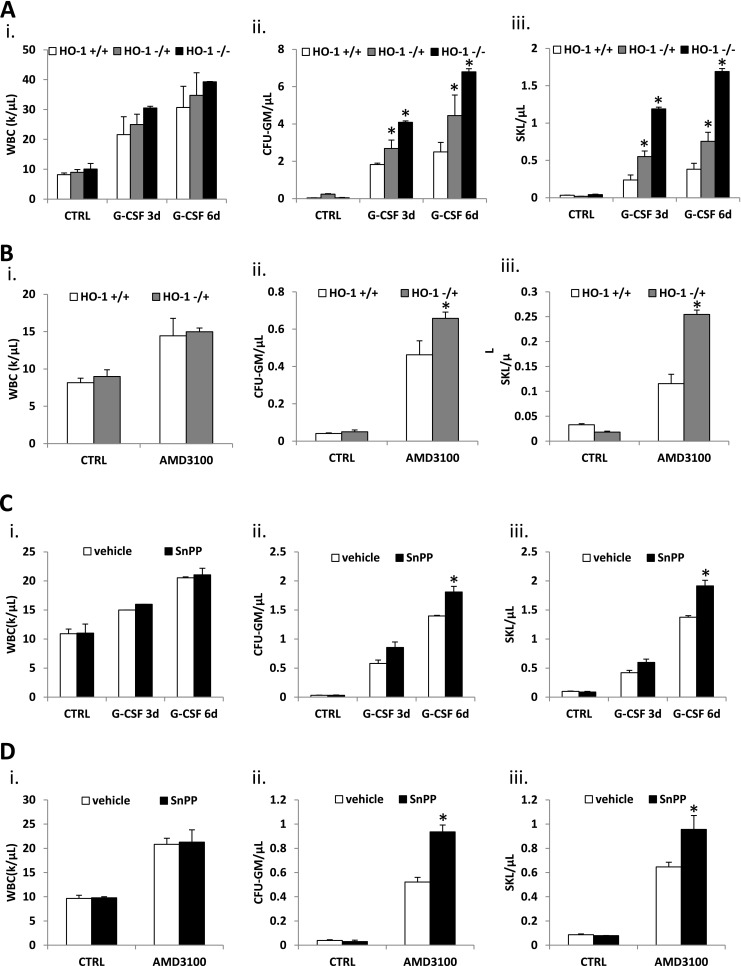
Loss of HO-1 function augments mobilization of bone marrow HSPC. The role of HO-1 in bone marrow stem cells mobilization was studied in HO-1 knockout mice (**Panel a and b**) and in wild type mice treated with HO-1 inhibitor SnPP (**Panel c and d**). Mice were mobilized with G-CSF for 3 and 6 days (100 μg/kg/day subcutaneously) (**Panel a and c**) and single dose of AMD3100 (5 μg/kg, intraperitoneally) (**panel b and d**). Mobilization was evaluated by number of circulating WBCs (*i*), clonogenic in vitro CFU-GMs (*ii*) and Sca-1^+^ c-kit^+^ Lin^−^ (SKL) cells by flow cytometry (*iii*) per microliter of peripheral blood. * *p* < 0.05. Data represent average of three independent experiments with at least four mice per experimental group

**Fig. 4:**
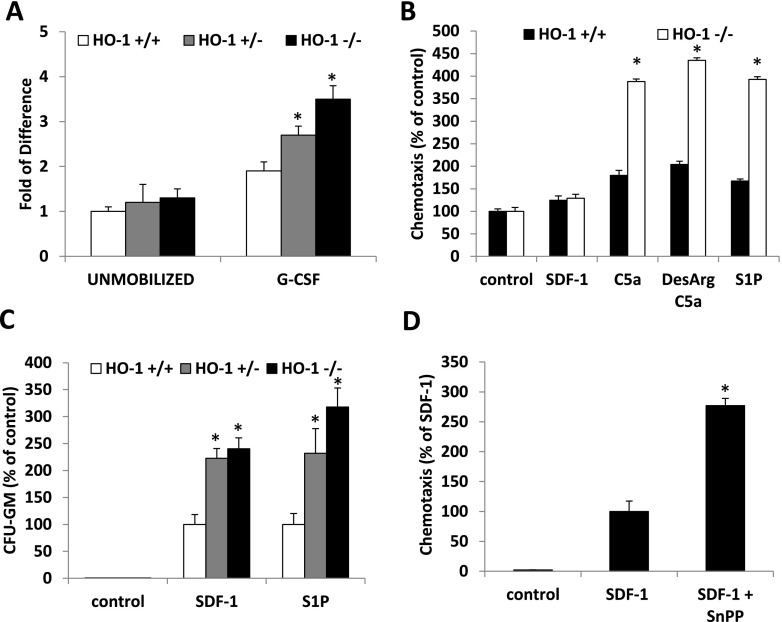
**Panel a**. Activation of ComC as measured by increase in concentration of C5a cleavage fragments in PB. Control values in non-mobilized WT mice were assumed to be 1.0. Mice were mobilized with G-CSF for 6 days (50 μg/kg/day subcutaneously). * *p* < 0.05 as compared to WT mice. **Panel b** – Chemotactic responsiveness of Gr-1^+^ granulocytes from control and HO-1-defcient mice. Chemotaxis to SDF-1 (300 ng/ml), C5a (120 ng/ml), _desArg_C5a (140 ng/ml) and S1P (0.1 μM) was perfomed with Gr-1^+^ cells purified from HO-1^+/+^ (WT), HO-1^+/−^ and HO-1^−/−^ mice and number of cells that migrated spontaneously to lower chambers containing medium only was assumed to be 100 %. * *p* < 0.05 as compared to WT mice. **Panel c**. HO-1 deficiency increases chemotactic activity of bone marrow HSPC. Chemotaxis to SDF-1 (300 ng/ml) or S1P (0.1 μM) gradient was performed with bone marrow mononuclear cells isolated from HO-1^+/+^ (WT), HO-1^+/−^ and HO-1^−/−^ mice and number of CFU-GM that migrated to SDF-1 or S1P in HO-1^+/+^ (WT) was assumed to be 100 %. Number of CFU-GM migrated to SDF-1 gradient was evaluated in clonogenic assay in methylcellulose cultures in presence of IL-3 (5 ng/mL) and GM-CSF (5 ng/mL). * *p* < 0.05. Data represent average of three independent experiments performed in triplicate. **Panel c**. HO-1 inhibition by SnPP enhances migration of normal CFU-GM to SDF-1 gradient. Number of CFU-GM that migrated to SDF-1 (300 ng/ml) gradient in absence or presence of SnPP (25 μM) was evaluated in clonogenic assay in methylcellulose cultures in presence of IL-3 (5 ng/mL) and GM-CSF (5 ng/mL). * *p* < 0.05. Data represent average of three independent experiments performed in triplicate. Migration of cells to SDF-1 gradient has been assumed to be 100 %

To evaluate BM retention of HSPCs in these animals under stress-directed conditions we performed direct mobilization studies employing G-CSF (Fig. 3a) and AMD 3100 (Fig. 3b) and noticed that HO-1-deficient mice easily mobilize HSPCs into PB according to the degree of HO-1 deficiency (HO-1^−/−^>HO-1^+/−^>WT). A similar effect was obtained in WT mice in which we inhibited HO-1 by i.p. administration of the HO-1 small-molecule inhibitor tin protoporphyrin IX (SnPP) (Fig. 3c and d). As reported in the past [10] mobilization of HSPCs into PB was not related to changes in plasma level of SDF-1 (Supplementary Figure 1A). Importantly, an enhanced mobilization of HSPCs in HO-1-deficient mice was correlated with enhanced activation of ComC in PB in response to G-CSF (Fig. 4a).

Several positive modulators of mobilization have been described [3–6], while little is known about pathways that negatively modulate this process. Our data for the first time identifies HO-1 as an important negative modulator of HSPC mobilization. Based on published literature we hypothesized that enhanced mobilization in HO-1-defcient mice is related to i) a decrease in SDF-1 level in BM stroma and thus impaired retention of HSPCs in BM niches [15], ii) a lack of anti-inflammatory effect of HO-1 [11] that leads to enhanced activation of mobilization promoting ComC and iii) enhanced motility of HO-1-defective cells [14].

In support of this, Gr-1^+^ neutrophils sorted from HO-1-defcient mice (Supplementary Figure 1B), that are well known as first cells that egress during BM during mobilization in order to pave a way for HSPCs across blood-BM barrier [9] showed enhanced chemotactic responsiveness to C5a and _desArg_C5a gradient (Fig. 4b). More important we demonstrate for a first time that also HSPCs from HO-1-deficient mice present enhanced migration toward SDF-1 and S1P gradient (Fig. 4c). These observations corroborate with report that HO-1 is negative regulator of neutrophil migration [14]. To support this further we performed Transwell migration assay and demonstrated that inhibition of HO-1 by non-toxic small-molecule inhibitor of HO-1 (SnPP) similarly as reported for neutrophils [14] significantly enhanced Transwell migration of HSPCs toward SDF-1 gradient (Fig. 4d).

Furthermore, since ComC plays a crucial role in mobilization of HSPCs [5, 8–10] and overexpression of HO-1 upregulates as reported expression of one of the ComC inhibitors, decay-accelerating factor (DAF or CD55) on the surface of erythrocytes [12], we explored a possibility that one of anti-mobilizing effects of HO-1 could be related to this effect. However, we did not observed any differences in expression of DAF/CD55 on surface of erythrocytes between HO-1 deficient and normal mice (data not shown).

As we have demonstrated here, HO-1-deficient mice are easy mobilizers, which could be at least partially the result of a decrease in SDF-1 level in BM stroma and defective retention of HSPCs in BM niches (Fig. 2). Interestingly, it has been reported [16] mice lacking one allele of HO-1 (HO-1^+/−^) showed accelerated hematopoietic recovery from myelotoxic injury, and HO-1^+/−^ HSCs repopulated lethally irradiated recipients with more rapid kinetics. This effect of accelerated hematopoietic recovery of HO-1^+/−^ mice can be explained by the possibility that defective retention of HSPCs prevents their niche-mediated quiescence in BM and may promote their expansion as demonstrated elegantly in mice transplanted with CXCR4 blocking agent [18].

**Fig. 5:**
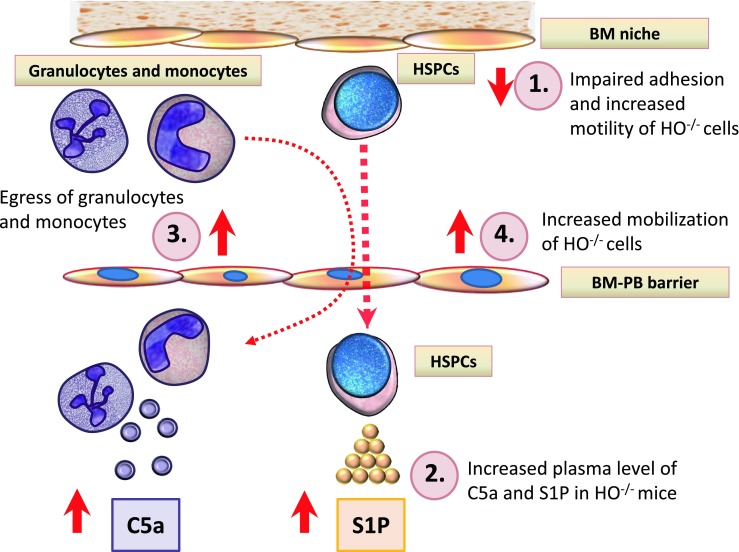
Proposed involvement of HO-1-deficiency in mobilization process of HSPCs. There are four levels at which HO-1-defciency may affect HSPC mobilization. **Level 1** - decrease in SDF-1-mediated BM retention signal for HSPCs as result of i) decrease in SDF-1 expression by BM stroma cells and ii) as result of an enhanced activation of ComC that leads to C5 mediated stimulation of granulocytes in the BM microenvironment. Granulocytes in response to C5a and _desArg_C5a secrete proteolytic enzymes that additionally perturb HSPCs retention signals (e.g., SDF-1-CXCR4 and VLA-4-VCAM-1 interactions). **Level 2** – hyperactivation of ComC results in increase in C5a and _desArg_C5a and S1P levels in blood plasma. **Level 3** - serum C5a and _desArg_C5a chemoattract from BM granulocytes that “pave the way” for HSPCs, which follow granulocytes and migrate through the endothelial barrier (“ice breaker phenomenon”) **Level 4** - HSPCs released from their niches respond to serum S1P and egress from BM. Finally, we cannot exclude the potential role of HO-1 on permeabilization status of the BM sinusoids endothelium, which will require further studies

In conclusion, our data demonstrate for the first time that HO-1 plays an important and heretofore unrecognized role in modulating positively SDF-1 levels in the BM microenvironment and thus plays a role in SDF-1-mediated retention of HSPCs in BM niches. What we also show here for first time is that HO-1 is a negative regulator of responsiveness of HSPCs to chemotactic gradients, and that HSPCs from HO-1 deficient mice respond robust to S1P that is a major chemoattractant for HSPCs in PB [10, 19]. HO-1 deficient mice are easy mobilizers and HO-1 negatively affects egress of HSPCs from BM at least partially by attenuating ComC activation what provides also further support for a pivotal role of ComC in mobilization of HSPCs. Our interpretation of HO-1 involvement in stem cell mobilization is graphically depicted at Fig. 5. Furthermore, our data showing a mobilizing effect by a non-toxic small-molecule inhibitor of HO-1 (SnPP) suggest that blockade of HO-1 would be a promising strategy to facilitate mobilization of HSPCs. Finally, based on enhanced responsiveness of HO-1 deficient cells to SDF-1 gradient further studies are also needed to understand better the molecular mechanisms responsible for the potential effect of HO-1 in homing of HSPCs after transplantation.

## Electronic supplementary material

Below is the link to the electronic supplementary material.

Supplementary Figure 1 (PPTX 160 kb)
