# Supplementary material for: Identification of Heme Oxygenase 1 (HO-1) as a Novel Negative Regulator of Mobilization of Hematopoietic Stem/Progenitor Cells
Source: Stem Cell Rev. 2014 Aug 3;11(1):110–8. doi: 10.1007/s12015-014-9547-7 (PMC4333311; doi:10.1007/s12015-014-9547-7)
Supplement: Supplementary file 1 — (PPTX 160 kb) [file 12015_2014_9547_MOESM1_ESM.pptx]

## Slide 1
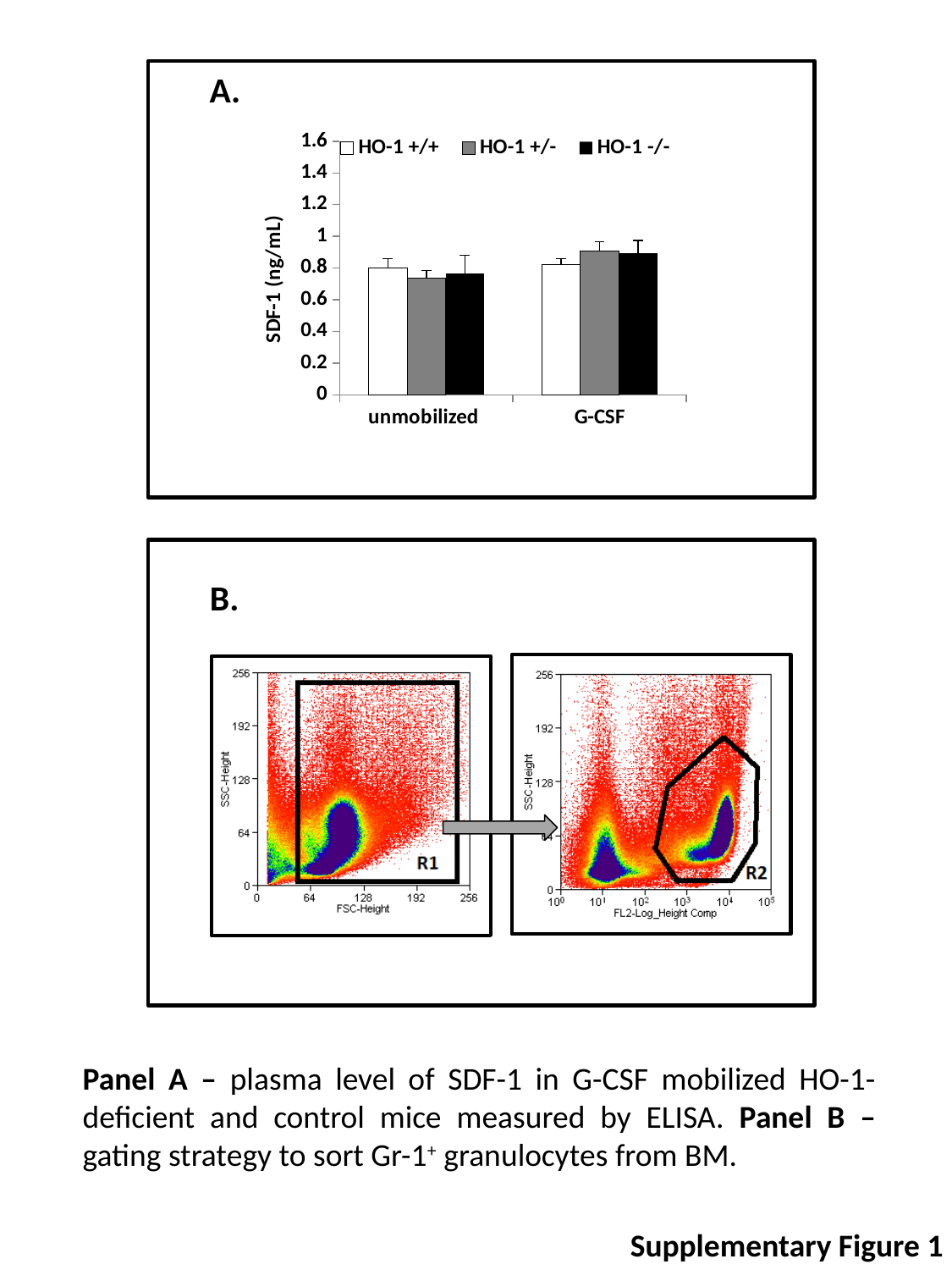

A.
### Chart
| Category | HO-1 +/+ | HO-1 +/- | HO-1 -/- |
|---|---|---|---|
| unmobilized | 0.8013089999999999 | 0.736338 | 0.7652140000000001 |
| G-CSF | 0.822966 | 0.909594 | 0.8951560000000001 |
B.
Panel A – plasma level of SDF-1 in G-CSF mobilized HO-1-deficient and control mice measured by ELISA. Panel B – gating strategy to sort Gr-1+ granulocytes from BM.
 Supplementary Figure 1
